# Cardiovascular Disease and the Mediterranean Diet: Insights into Sex-Specific Responses

**DOI:** 10.3390/nu16040570

**Published:** 2024-02-19

**Authors:** Anushriya Pant, Derek P. Chew, Mamas A. Mamas, Sarah Zaman

**Affiliations:** 1 Westmead Applied Research Centre, Faculty of Medicine and Health, University of Sydney, Sydney, NSW 2145, Australia; anushriya.pant@sydney.edu.au; 2 Victorian Heart Hospital, Victorian Heart Institute, Monash University, Melbourne, VIC 3800, Australia; 3 Keele Cardiovascular Research Group, Keele University, Newcastle ST5 5BG, UK; 4 Department of Cardiology, Westmead Hospital, Sydney, NSW 2145, Australia

**Keywords:** cardiovascular disease, prevention, diet, Mediterranean diet, sex-specific, women’s health, pregnancy complication, polycystic ovarian syndrome, review

## Abstract

Cardiovascular disease (CVD) is a leading cause of mortality and disease burden in women globally. A healthy diet is important for the prevention of CVD. Research has consistently favoured the Mediterranean diet as a cardio-protective diet. Several studies have evaluated associations between the Mediterranean diet and cardiovascular outcomes, including traditional risk factors like hypertension, type 2 diabetes mellitus, and obesity. In addition, consistent evidence suggests that the components of the Mediterranean diet have a synergistic effect on cardiovascular risk due to its anti-inflammatory profile and microbiome effects. While the benefits of the Mediterranean diet are well-established, health advice and dietary guidelines have been built on largely male-dominant studies. Few studies have investigated the beneficial associations of the Mediterranean diet in sex-specific populations, including those with non-traditional risk factors that are specific to women, for instance polycystic ovarian syndrome and high-risk pregnancies, or more prevalent in women, such as chronic inflammatory diseases. Therefore, this review aims to provide a comprehensive overview of the current evidence regarding the Mediterranean diet in women in relation to cardiovascular health outcomes.

## 1. Introduction

Cardiovascular disease (CVD) is the main cause of mortality in women globally, responsible for 35% of all female deaths in 2019 [1]. Nutrition plays an important role in substantially modifying cardiovascular risk factors and reducing the risk of developing CVD [2]. Historically, different types of diets have been advocated for both the primary and secondary prevention of CVD [3]. The Mediterranean diet (MD) is internationally recognised as the most recommended and widely established diet [4]. This recognition emerged in the 1960s with the “Seven Countries Study” that investigated dietary behaviours and health outcomes globally and observed a lower incidence of CVD in the Mediterranean populations of Italy and Greece [5,6]. The MD is based on the traditional dietary patterns of those historically from the Mediterranean basin, characterized by a high consumption of plant foods (such as fruit, vegetables, wholegrains, legumes, and nuts) and mono-unsaturated fats from extra-virgin olive oil (EVOO), moderate consumption of dairy and fish/seafood, and low consumption of red and processed meats [4,7].

Several meta-analyses have demonstrated a beneficial effect of the MD on CVD risk and cardiovascular risk factors [8,9,10,11]. The first meta-analysis looking specifically at women and CVD risk was published only recently, in 2023 [11]. Pant et al. [11] found that higher versus lower MD adherence was associated with a 24% lower risk of incident CVD and a 23% lower risk of premature mortality. 

Sex differences are important to consider when delivering nutritional advice for the prevention of CVD. Women have been under-represented and under-studied in dietary trials, and most cardiovascular research has been conducted in predominantly male populations [1,12]. In addition, women can have non-traditional CVD risk factors, for example, past pregnancy conditions (such as pre-eclampsia and gestational diabetes (GDM)), polycystic ovarian syndrome (PCOS), and female-predominant conditions like inflammatory autoimmune diseases (such as rheumatoid arthritis (RA) and systemic erythematous lupus (SLE)) can contribute to CVD [13,14]. Therefore, there is a need for targeted nutrition research in female-specific populations and to synthesize work that enables tailored CVD guidelines for women with non-traditional risk factors [15,16]. This review aims to summarize the current evidence on the MD for the prevention of CVD in female-specific populations. 

## 2. Cardio-Protective Benefits of the MD

Several mechanistic pathways have been associated with the beneficial effects of the MD on cardiovascular outcomes. Previous research has suggested that the MD has cardio-protective advantages due to a synergistic effect of its main food components, including key nutrients and food groups [6]. These include omega-3 poly-unsaturated fats from fish and unsaturated fats including EVOO, polyphenols, antioxidants, and increased fibre content from plant foods [6]. 

Atherosclerosis is a common characteristic across CVD pathologies, resulting from a gradual process of lipid accumulation, inflammation, and endothelial dysfunction [17]. These factors lead to the formation of fatty streaks that can be followed by immune-mediated plaque formation in the coronary vessel wall and, potentially, plaque rupture [17]. The MD has been associated with the following cardio-protective effects: improved lipid profile, improved vascular function and blood pressure (BP), and reduced oxidative stress and inflammatory biomarkers that may have antioxidant and anti-atherosclerotic effects [18,19]. 

**Figure 1:**
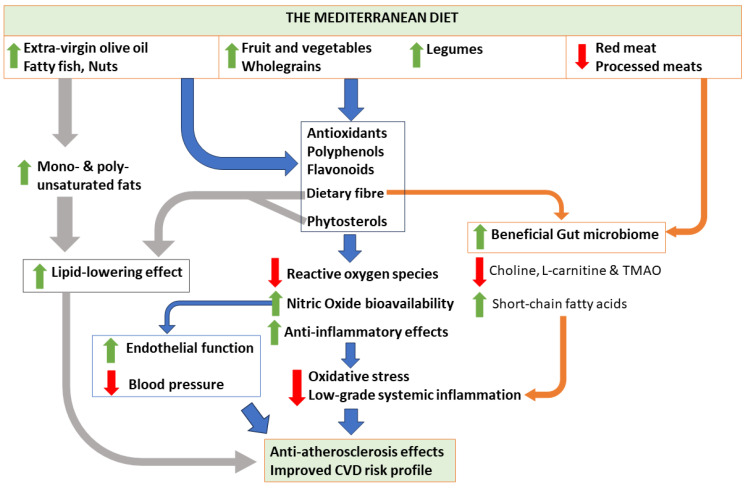
Possible mechanisms associated with the Mediterranean diet and its components, and their effects on cardiovascular health. TMAO, trimethylamine N-oxide; CVD, cardiovascular disease.

Tosti et al. [20] summarized the main factors that are important in mediating the impact of the MD. An interplay between these pathways may explain this diet’s favourable effects on CVD (Figure 1). Firstly, studies have suggested that the MD has a lipid-lowering effect due to a higher intake of mono- and poly-unsaturated fats from plant sterols and fish [20]. Additionally, the increased consumption of dietary fibre and phytosterols may help to mediate cholesterol absorption in the gut [20]. Secondly, the MD is also rich in antioxidants, flavonoids, and minerals due to high consumption of plant foods and EVOO [20]. These nutrients have anti-inflammatory effects and reduce oxidative stress [20]. The increase in antioxidants may also lead to a reduction in reactive oxygen species (ROS), while enhancing the bioavailability of nitric oxide and therefore improving vascular function and BP [6,21]. Thirdly, accumulating evidence highlights the importance of the gut microbiome and the impact of a healthy diet on host biology [22]. In particular, the MD was reported to have an over 50% lower content of choline and L-carnitine than the Western diet [20,23]. Both are found in red meat and have been shown to increase the risk of CVD through the production of the pro-thrombotic pro-inflammatory trimethylamine N-oxide in the gut [20,23]. Further, dietary fibre has beneficial effects on the gut microbiota by generating short-chain fatty acids, which eventually leads to reduced low-grade inflammation [24].

## 3. Comparison with Other Diets

### 3.1. Western Diet

The Western diet has been described as the common denominator for many non-communicable conditions [25,26]. This diet has been associated with a higher intake of unhealthy fats, refined carbohydrates, sodium, and ultra-processed foods, while being low in dietary fibre and micronutrients [25]. The poor nutritional balance found in the Western diet is thought to alter the gut microbiome and increase both local and systemic inflammation [27]. On the other hand, the MD offers a whole-foods, nutrient-rich approach with foods that are higher in fibre, omega-3 poly-unsaturated fats, and antioxidants, all contributing to its anti-inflammatory and vaso-protective effects [28]. 

### 3.2. Low-Fat Diet

In addition to the MD, several healthy dietary patterns have been identified for the prevention of CVD [29]. These diets are generally characterized by their similar nutrient profiles and individual food components, such as unrefined whole grains, high dietary fibre and antioxidants from fruits and vegetables, and lower intake of red/processed meats (Table 1) [30].

In contrast to the Western diet, the low-fat diet has been associated with cardiovascular health benefits, especially for weight loss [44]. Low-fat diets consist of no more than 30% fat, and some studies have suggested a lower fat intake of 10–15% [45]. Both the low-fat diet and the MD emphasize fresh, minimally processed plant foods such as vegetables, fruits, whole grains, legumes, and fish. However, the MD is differentiated from the low-fat diet due to its higher mono- and poly-unsaturated fat intake from EVOO, nuts, and fatty fish [33]. These nutrients are also rich sources of antioxidants and polyphenols that help improve the lipid profile, inflammatory biomarkers, and endothelial function [6]. Many comparative studies have shown that the MD, compared to the low-fat diet, is associated with greater decreases in body weight, body mass index (BMI), BP, blood glucose, and total cholesterol [31,32,33].

### 3.3. Dietary Approaches to Stop Hypertension (DASH)

The DASH dietary pattern was first created to reduce BP in patients with hypertension. It has been associated with many cardiovascular benefits to CVD, BP, and lipid regulation [46,47]. Like the MD, the DASH diet focuses on a higher intake of plant foods and a lower intake of dairy and red meat [47]. Both diets lead to anti-inflammatory effects, increased antioxidant levels, and reduced glycaemic load [34]. The DASH diet differs from the MD, with a lower fat content (~27%) and additional advice on limiting sodium [34]. The DASH diet is notably high in minerals from the high consumption of fruits and vegetables that have been associated with anti-hypertensive properties, such as potassium, magnesium, and calcium [34].

Studies comparing the two diets are very limited and conflicting [34,35,36,37]. A 2021 prospective cohort study found that higher MD adherence was associated with a 10-year reduced risk for CVD, but showed no significance of the DASH diet with regard to the CVD risk [34]. Similarly, another cohort study found a significant inverse association only for the MD diet, with sudden cardiac death [35]. 

Smaller studies have also observed that the diets have differing effects on the cardiovascular risk factors. A cross-sectional study found that the DASH diet was associated with improvements in different lipids (total cholesterol, triglycerides, low-density lipid cholesterol (LDL-C), high-density lipoprotein cholesterol (HDL-C), and LDL/HDL ratio), while the MD only improved the LDL/HDL ratio [36]. Another study found that both diets were inversely associated with diastolic BP and fibrinogen levels, while adherence to the DASH diet further improved insulin levels and high-sensitivity C-reactive protein (hs-CRP), an inflammatory biomarker [37].

### 3.4. Vegetarian/Vegan Diets

Plant-based dietary patterns, such as vegetarian or vegan diets, have emerged in recent decades as being cardio-protective [48]. These diets have been associated with favourable effects on lipid profile, weight loss, glycaemic control, and cardiovascular mortality [48,49]. Plant-based diets are generally characterized by a high consumption of plant foods, depending on the type of vegetarianism. For example, vegan diets omit all animal products, while lacto-ovo vegetarian diets allow animal by-products (e.g., eggs, dairy, and honey). 

Both the MD and the plant-based diets focus on consuming more plant foods and less saturated fat from red/processed meats compared to the Western diet. The main difference is the lack of seafood-derived omega-3 fatty acids in plant-based diets. 

Few studies have compared the MD and plant-based diets and reported similar cardiovascular benefits [38,39,40]. For example, the 2018 Cardiovascular Prevention with Vegetarian Diet (CARDIVEG) Study found that both diets led to significant reductions in BMI, fat mass, and body weight, but no group differences [38]. This contrasts with the randomized crossover trial of Barnard et al. [40], which found significant group differences between the vegan diet and the MD. For example, the vegan diet was associated with improvements to metabolic parameters like insulin resistance, body weight, and lipid concentrations. On the other hand, the MD was more beneficial to BP [40].

### 3.5. Region-Specific Dietary Patterns 

Region-specific dietary patterns have gained interest in recent years, including the Nordic diet as another traditional diet with comparable cardiovascular health effects to the MD [29]. The Nordic diet follows the regional eating habits of Nordic countries of Denmark, Finland, Iceland, Norway, and Sweden. Like the MD, the Nordic diet encourages a higher intake of plant foods (vegetables and fruits), whole grains (e.g., rye bread), legumes, and fatty fish [50,51]. In addition, the Nordic diet promotes intake of low-fat dairy products and vegetable fats like rapeseed oil, berries, and root vegetables [51]. 

The notable difference is the choice of oil between these two diets. However, both EVOO and rapeseed oil provide similar cardio-protective properties from mono- and poly-unsaturated fatty acids. Rapeseed oil has higher levels of α-linolenic acid (ALA), phytosterols, and trace elements (e.g., ubiquinone) that have been linked with reductions in peroxidative damage [51]. The Nordic diet has been associated with a lower risk of CVD and significant decreases in total cholesterol, body weight, and diastolic BP [49,51]. Comparison studies investigating the effects of the Nordic diet and the MD on cardiovascular health are scant. A cohort study looking at body weight reported that neither the MD nor the Nordic diet was associated with significant changes [43]. Others have demonstrated that only the MD lowered the risk of type 2 diabetes mellitus (T2DM) and all-cause mortality [41,42]. 

Another region-specific dietary pattern is the traditional Japanese diet, characterized by increased vegetables, fruits, and seafood, and a lower intake of meat/dairy products [29]. In contrast to the MD, key Japanese foods in this diet include the high use of rice bran oil, soy products, green tea, seaweed, and fermented foods, such as miso soup and pickles [52]. Like the MD, adherence to a Japanese-style traditional diet has been associated with positive effects on CVD mortality, as demonstrated in a recent 2022 meta-analysis of cohort studies [29,52]. To date, no study has compared these two diets in terms of their cardiovascular benefits.

While the health benefits of the MD have been extensively studied, comparative studies with other healthy dietary patterns are lacking. Current evidence indicates that the MD may have similar effects to other diets, and some studies suggest that the MD is more effective for overall cardiovascular health. However, the efficacy of different heart-healthy diets compared with the MD has not been sufficiently evaluated. Studies that have compared MD with other diets are often limited by smaller sample sizes and short follow-up duration. This emphasizes the need for more robust studies that can be translatable into clinical practice.

## 4. Sex-Specific Mechanisms in Nutrition 

Biological sex is an important factor in cardiovascular research when considering cardiovascular outcomes at the population level [53]. Sex-specific interactions with diet may be influenced by differences in pathophysiology, hormones, and nutrient metabolism [13]. However, mechanistic interactions between sex and the MD are unclear, and studies looking at this relationship are limited. 

A 2023 pilot study reported sex differences in the host response to the MD [54]. Females adhering to the MD presented upregulation of the apolipoprotein E (APOE) gene and angiotensin-converting enzyme (ACE) expression compared to males [54]. The APOE gene is a pleiotropic protein that plays an important role in lipid metabolism through the removal of cholesterol and triglycerides from the bloodstream [54]. APOE binds to receptors on the liver to help facilitate the clearance of triglyceride-rich lipoproteins from the bloodstream [54]. ACE is involved in the regulation of BP within the renin–angiotensin–aldosterone system [54]. Both are important in the pathophysiology of cardiovascular risk factors [54]. 

Moreover, in vivo studies have reported sex-dimorphic responses in oxidative stress and inflammatory mechanisms [55]. For example, in mammalian tissue cultures, males have demonstrated a weaker host response, with a greater increase in ROS production and basal inflammation than females [55]. Males also presented a lower level of antioxidants that are critical in alleviating oxidative damage [55,56]. However, Bedard et al. [57] found that the effects of a four-week isocaloric MD intervention on systematic inflammation were similar in both sexes.

Animal models can help us to understand these interactions and sex-specific responses to dietary interventions [58]. Several animal studies have demonstrated that sex differences persist in nutrient metabolism, hormonal interactions, and the gut microbiome [58]. For example, one study found that a high-fat diet led to more weight gain and insulin resistance in male mice, while female mice had a higher abundance of beneficial microbes [59]. Another study found that female mice fed a high-fat diet were protected against low-grade systemic inflammation, while male mice presented with tissue inflammation and glucose intolerance [60]. However, no animal model has focused on the sex-specific responses to the MD or their effects on cardiovascular health [58]. 

The relationship between sex and dietary patterns may be explained by evolutionary pressures on dietary behaviours, physiology, and nutritional requirements [61]. Sex-specific dietary behaviours have been observed since the pre-Neolithic hunter–gatherer period [61]. Women often gathered fruit and vegetables, while men hunted and consumed more animal protein and high-fat foods to meet energy demands [61]. Hence, biological sex and prehistoric gender roles may have led to the modification of energy requirements and nutrient metabolism in both sexes [60,62]. Additionally, in cross-sectional studies, female participants have demonstrated better adherence to healthy diets than males [61]. For example, women have been shown to consume higher dietary fibre from fruits and vegetables, less fat, and lower-energy foods, but more sugary foods [61,63]. These sex-specific dietary behaviours, as well as biological and social factors, may contribute to sex differences in both male and female physiology, including the gut microbiome and metabolic pathways [64]. It could be postulated that evolutionary changes to biological sex- and gender-based dietary behaviours have resulted in men and women responding differently to diets [64].

## 5. Current Evidence on the MD and Cardiovascular Health

Most dietary studies in cardiovascular research have been observational (Table 2). There is consistent evidence favouring the cardio-protective effect of the MD [10]. Recent meta-analyses have demonstrated its beneficial effects for both the primary and secondary prevention of CVD [10,65,66,67,68]. Since the diet’s recognition in the 1960s [5], there have been several randomized controlled trials (RCT) on cardiovascular health, and a few on CVD [31,32,69]. While the effects of the MD on primary prevention have been investigated, there is a paucity of research on secondary prevention [32]. These studies have demonstrated that increasing adherence to the MD is associated with a lower risk of CVD, including the Prevención con Dieta Mediterránea (PREDIMED, Spain) for primary prevention [31], the CORonary Diet Intervention with Olive oil and cardiovascular PREVention (CORDIOPREV, Spain) [32], and the Lyon Diet Heart Study (France) for secondary prevention [69].

### 5.1. The MD and Cardiovascular Outcomes in Women Versus Men 

One of the largest prospective cohort studies on the MD in women was the Nurse’s Health Study (NHS) from the United States of America (US), which followed over 74,000 women (aged 30 to 55 years) for 20 years [70]. Fung et al. [70] found that higher adherence to the MD was associated with a 29% lower risk of coronary heart disease (CHD) [70]. Cohort studies have demonstrated a similar efficacy of the MD in reducing the risk of CVD in women and men [89,90,91], while others have reported no significance in terms of cardiovascular outcomes in women [92,93,94,95]. Some studies have suggested that the MD is associated with a more pronounced effect on CVD risk reductions in men than women [42,96], and few studies have reported significant effects of the MD on CVD risk only in women, and not in men [96,97]. From these sex-disaggregated individual studies, risk reductions for CVD ranged from 19% to 38% in women and 17% to 31% in men [11]. In mixed-sex cohorts, the percentages of women and men were mostly proportionate, and few had a higher proportion of females (>60%) [42,92,93].

However, older landmark RCTs on the MD and CVD have failed to report sex-disaggregated studies and tended to recruit a lower proportion of females. In 1999, the Lyon Heart Study (N = 605; 90% male participants) was a single-blinded secondary prevention trial that reported a 65% lower risk of cardiovascular deaths and non-fatal myocardial infarction with the MD [69]. The study’s authors did not report sex-disaggregated results, and recruited just 10% women. This limits the translation of this research into clinical practice for the female population.

Notably, the 2018 revised PREDIMED Study (N = 7447; 43% male participants) was a large, multi-centre, three-arm RCT that found a reduction in cardiovascular risk of 31% with an MD with EVOO and 28% with a MD with nuts compared to a low-fat diet [31]. It is the most well-known dietary trial, and over half of those included were female participants. Additionally, the authors performed subgroup analysis by sex and found that the combined effects of the MD groups were significant in men only, and not in women [31].

More recently, the 2022 CORDIOPREV study focused on patients with CHD (N = 1002; 82.5% male participants), who were assigned to either a MD or a low-fat group [32]. In this single-center RCT, Delgado-Lista et al. [32] found a 28% reduction in the risk of developing recurrent major cardiovascular events in those following the MD compared to a low-fat diet. Interestingly, the effects were more evident in men than women, with no statistical differences in women [32]. However, this study was limited by the smaller proportion of female participants, which likely contributed to its reduced statistical power and the insignificant result in women. 

Two sex-disaggregated meta-analyses on primary prevention with the MD have demonstrated a beneficial effect on CVD in both men and women; overall risk reductions ranged from 15% to 24% [10,11]. For secondary prevention, only one meta-analysis has been conducted, reporting a significant effect of the MD on total mortality in men only, but not in women [98].

### 5.2. Hypertension (or High BP)

Several studies have demonstrated the beneficial effect of the MD on BP [65,99,100]. Many of these individual studies have integrated the MD into a lifestyle intervention [65,99,100]; however, few have focused only on women [71,72,74,75,76,101]. In addition, these findings are inconsistent in sex-disaggregated analyses. Storniolo et al. [73] performed sex-stratified secondary analyses on a cohort of women with hypertension [N = 90] who were recruited from the PREDIMED study. In this study, women who consumed an MD, supplemented with either EVOO or nuts, demonstrated an improved expression of endothelial markers and lower BP [73]. In contrast, a Canadian MD intervention (N = 70; 54.3% male participants) found a reduction in systolic BP in males only [102]. However, this study found no significant interaction with sex and may possibly have been limited by the small sample size. This is consistent with the findings of Jennings et al. [77] in another sex-disaggregated analysis (N = 1294; 56.7% male participants), which demonstrated that a Mediterranean-style diet led to a significant decrease in systolic BP in males, but not in females [77]. Jennings et al. [77] also found significant improvements in arterial stiffness, an important predictor of developing hypertension, in female participants only. 

### 5.3. T2DM

The MD has been significantly associated with a lower risk of T2DM and improvements in blood glucose and glycated haemoglobin levels [72,103,104,105]. In women, both randomized and observational cohort studies have reported a beneficial effect of following the MD on lowering the incidence of T2DM and improving metabolic and insulin resistance biomarkers [78,106]. A 2020 prospective cohort study, including over 25,000 women from the Women’s Health Study (WHS, US) who were followed for 20 years, found that higher MD adherence was associated with a 30% lower risk of future T2DM [78]. This is consistent with other similar non-sex-specific cohort studies [103,104]. In another analysis using the same cohort of women, Ahmad et al. [94] found that parameters that mediated the inverse association between MD adherence and CVD risk included biomarkers of glucose metabolism and insulin resistance, both of which are important in the pathophysiology of T2DM. 

Two PREDIMED subgroup analyses have reported sex-disaggregated findings [79,107]. In nondiabetic participants (N = 418; 41.6% male participants), Salas-Salvado et al. [79] found that the MD groups supplemented with EVOO/nuts were inversely associated with incident T2DM (risk reductions by 51–52%) in non-sex-disaggregated analyses [79]. However, in sex-stratified data, the authors reported a significant effect of the MD groups on T2DM risk reduction in female participants only [79]. Another PREDIMED subgroup analysis (N = 772; 43.9% male participants) reported a beneficial effect of the MD on plasma glucose levels, with no significant interactions with sex [107].

### 5.4. Anthropometric and Metabolic Parameters 

The MD has been associated with other cardiovascular risk factors, including its effectiveness on weight loss and improvements in the lipid profile [75,108,109]. However, few studies have reported sex-disaggregated results. In a four-week isoenergetic intervention based in Canada, which included men and premenopausal women (N = 70; 54.3% male participants), MD adherence was associated with lower body weight, BMI, total cholesterol, LDL-C, and 2-h post-load insulin [102]. Men, compared to women, adhering to the MD had greater decreases in body weight and BMI [102]. A similar 12-week combined lifestyle intervention (high-intensity intermittent exercise, MD regime, and fish oil) based in Australia was conducted in premenopausal women who were overweight (N = 30) [74]. This study found that the Mediterranean lifestyle intervention resulted in significantly lower BMI, body fat, and central adiposity compared to the control group, and improvements to metabolic parameters such as fasting plasma insulin and inflammatory marker interleukin-6 were also observed [74]. No significant changes were seen with lipids [74]. 

In postmenopausal women with T2DM (N = 279), an integrated Mediterranean Lifestyle Program (MLP) from the US was conducted over 6 months, with weekly meetings on self-management and guidance following the MD, exercise, and smoking cessation [72]. The study demonstrated that the MLP led to positive effects on BMI and plasma fatty acids [72]. More recently, in a 2018 RCT of postmenopausal women with central obesity (N = 144) from Finland, both the MD and the low-fat diet showed similar effects on anthropometric and lipid parameters [76].

## 6. The MD and Female-Specific Conditions

It is critical to have a tailored and customised approach to the treatment and prevention of CVD, and to reduce disease burden in women with female-specific (or so-called “non-traditional”) risk factors. These female-specific conditions are apparent throughout a woman’s reproductive life and include the following: PCOS, pregnancy complications (for example, GDM, hypertensive disorders of pregnancy (HDP), or preterm delivery), and female-predominant inflammatory auto-immune conditions such as RA and SLE.

### 6.1. PCOS

PCOS is one of the most prevalent endocrine conditions in reproductive premenopausal women, and lifestyle modification is the main treatment to control its symptoms [110]. Additionally, the impact of PCOS on the future risk of developing CVD is well known and involves numerous cardiometabolic conditions, such as insulin resistance, dyslipidaemia, and hypertension [111]. 

In a 12-week RCT (China), 72 patients with PCOS and who were overweight were randomized to either the low-carbohydrate MD or the low-fat diet [80]. The low-carbohydrate MD resulted in significantly higher reductions in anthropometric parameters (body weight, BMI, and body fat percentage) and metabolic parameters, such as fasting insulin, blood glucose levels, and insulin resistance index [80]. However, changes in the lipid profile were more prominent in the low-fat group, with lower LDL-C, total cholesterol, and triglycerides [80]. Another RCT (N = 144) based in the US investigated the beneficial effects of two hypocaloric dietary interventions, the MD versus the ketogenic diet [81]. Cincione et al. [81] found that both groups had significant changes in anthropometric and metabolic parameters, but the ketogenic diet was more effective in terms of reducing BMI and insulin resistance [81].

The MD for the primary prevention of PCOS has been investigated in case–control and cross-sectional studies, which have reported inconsistent results. Higher adherence to the MD has not been associated with a lower risk of developing PCOS [112,113,114]; however, this may be related to confounding factors. In contrast, women diagnosed with PCOS were more likely to consume an MD compared to those without PCOS [115].

### 6.2. Pregnancy-Related Complications

Past pregnancy-related complications are well-established, non-traditional risk factors for CVD. Several meta-analyses have indicated a nearly twofold increase in the chance of developing a future cardiovascular event in those with GDM, HDP, or preterm delivery [88]. Pregnancy involves complex interactions between the mother and the foetus, with physiological changes in the woman’s body as a means of supporting the growth of the foetus [116]. While the pathophysiology of pregnancy complications is unclear, it can be hypothesized that pregnancy alone may drive many abnormal biological alterations that predispose women to cardiometabolic diseases in later years, including impaired placentation, abnormal haemodynamics, and endothelial dysfunction [117]. Pre-pregnancy environmental factors may also influence the health status during pregnancy, for example, higher BMI, maternal nutrition, stress levels, and smoking [118,119].

Healthy maternal nutrition has been well-studied during the prenatal period and during pregnancy to prevent adverse pregnancy outcomes [118]. Among women with inadequate nutrition, nutritional supplementation and tailored dietary advice are associated with better birth outcomes [118]. This includes having a diet based on a nutrient-dense, whole-foods approach, like the MD. Short-chain poly-unsaturated fatty acids (LC-PUFA), predominantly found in MD foods like fatty fish and nuts/seeds, are considered essential during pregnancy, and a deficiency may be linked to adverse pregnancy complications like pre-eclampsia and preterm delivery [120,121,122,123]. Omega-3/6 LC-PUFA regulates various physiological and pathological pathways associated with normal growth and function of the placenta, as well as maternal health [123]. This includes playing a key role in vascular remodelling during pregnancy and regulating inflammation to prevent pregnancy complications such as HDP [123].

Additionally, the 2020 review conducted by Traylor et al. [124] investigated the link between maternal stress and adverse pregnancy outcomes, suggesting the use of lifestyle-related interventions for stress reduction during pregnancy. Chronic stress and anxiety have been suggested to alter maternal homeostasis and foetal development by activating the hypothalamo–pituitary–adrenal (HPA) stress response and therefore increasing inflammatory activity [124,125]. However, studies evaluating the effects of lifestyle interventions on stress and pregnancy outcomes are lacking. In an RCT of 1221 individuals (Spain, 100% female participants) with high-risk pregnancies, Crovetto et al. [84] conducted a parallel RCT (Improving Mothers for a better PrenAtal Care Trial BarCeloNa—IMPACT BCN) with two structured interventions: the MD or a mindfulness-based stress reduction. The authors found that both interventions led to a significantly reduced risk of SGA babies after delivery. More recently, in 2023, a secondary analysis from the IMPACT-BCN trial reported that the MD, compared to the usual care, was beneficial in terms of reducing maternal stress and improving overall sleep quality during pregnancy [85]. 

Lifestyle modification is the primary approach to reducing cardiovascular risk in these women [116,126]. While many have investigated the MD for the prevention and treatment of GDM or HDP [86,127,128], there are few dietary interventions [129,130] that have assessed the postpartum period, and only two MD interventions [82,83]. In a Spanish cohort of 260 women with past GDM, Perez-Ferre et al. [82] conducted a 2-h group intervention that focused on the Mediterranean lifestyle and found that the intervention group had reduced incidence of impaired glucose intolerance. The study also reported that women with past GDM in the MD intervention presented with significantly lower levels of triglycerides and LDL-C compared to the control. Reimer et al. [83] (N = 38) found that a nutritional 6-month intervention with an MD, based in Germany, led to significant reductions in systolic and diastolic BP in women with past HDP [82]. Moreover, a 2023 meta-analysis of six studies in women with past HDP suggested that the current dietary interventions are in need of re-designing due to their limited generalizability and lack of standardized outcomes [83].

### 6.3. Female-Predominant Inflammatory Autoimmune Conditions

Autoimmune diseases are a diverse range of conditions [131] that have different manifestations and varying ages of onset, with most autoimmune conditions, such as RA and SLE, occurring in women [131]. While the exact mechanism is not well understood, it has been hypothesized that there is a genetic susceptibility to autoimmune diseases in women compared to men due to the X chromosome [131,132]. These chronic inflammatory conditions are associated with an increased risk of CVD in both sexes [13]. However, considering the female predominance of autoimmune disorders, these conditions tend to be more prevalent risk factors for CVD in women [13].

The effects of the MD on the prevention and treatment of RA have been studied [133]. RA is typically characterized by inflammation of the joints, and the MD may provide additional benefits to minimize symptoms, including joint pain and physical function [133]. The MD is commonly known for its anti-inflammatory effects and higher content of antioxidants, which may have protective effects in people with RA [134]. McKellar et al. [135] (Scotland) conducted a pilot study of 130 women with RA (aged 30 to 70 years) who were recruited into a Mediterranean-style diet intervention over six weeks, and found improvements in systolic BP. More recently, in 2023, Papandreou et al. [87], from Greece, conducted a 12-week lifestyle intervention with an integrated MD and physical activity program in 40 female participants (aged ≥18 years) with mild-to-moderate RA. This study found that the integrated MD lifestyle program significantly improved body weight, BMI, and blood glucose, but not lipid profile [87].

In women with SLE (N = 58), a 12-week aerobic training intervention found no significant group differences in outcomes of body composition, BMI, or MD adherence [136]. This contrasts with a female-majority cross-sectional study of 280 patients with SLE (90.4% female participants) based in Spain, that found greater MD adherence to be associated with decreased SLE disease activity and improvements in BMI, fat mass, and triglycerides [88].

While there is emerging sex-specific knowledge on CVD, the current dietary advice is limited due to a lack of research investigating the MD for CVD prevention in these different subgroups of women.

## 7. Future Directions

Future cardiovascular research is required in order to increase the representation of women and to develop dietary trials that report female-specific, sex-disaggregated data. Emerging studies should aim to increase recruitment of female participants in both primary and secondary prevention trials. Further work may consider focusing on female-specific populations that examine the MD and/or its different components. More lifestyle interventions with an MD are needed for women during the postpartum period and those with female-specific risk factors, including PCOS.

## 8. Conclusions

The current evidence within this review supports the benefits of the MD across different populations, including both sexes and women at high risk of CVD. However, more research is needed in order to postulate whether the MD is more beneficial than other contemporary diets for female-specific conditions. While recent CVD guidelines have considered sex-specific risk assessment, dietary advice for the prevention of CVD lacks a tailored approach for women.

## Figures and Tables

**Table 1:** Comparison of the Mediterranean diet with other heart-healthy dietary patterns.

Diet	Foods	Cardio-Protective Properties	Cardiovascular Benefits	Comparative Studies with MD	Results: MD vs. Comparator Diet
LF	Vegetables, fruits, whole grains, legumes, lean meats, seafood/fish, low-fat dairy <30% fat intake of total energy	Lipid-lowering effects on total and LDL cholesterol levels	Weight loss Better lipid profile Lower risk of CVD	Estruch et al., 2018 [31], Spain (RCT) Delgado-Lista et al., 2022 [32], Spain (RCT) Nordmann et al., 2011 [33], (SR)	Adherence to MD vs. LF led to: Lower incidence of MACE Greater changes in body weight and BMI Greater reductions in DBP and SBP Greater reductions in inflammatory marker hs-CRP
DASH	Vegetables, fruits, whole grains, legumes, low-fat dairy, and nuts Limit intake of cholesterol, total/saturated fat, and red/processed meats Limited added sugars Sodium intake restricted to 1500 mg/d	High in dietary fibre and protein Increased intake of minerals associated with reducing BP, like potassium and magnesium	Reduced SBP and DBP Better lipid profile Lower risk of CVD and T2DM	Critselis et al., 2021 [34], Greece (Prospective cohort) Bertoia et al., 2014 [35], US (Prospective cohort) Panbehkar-Jouybari et al., 2021 [36], Iran (Cross-sectional) Jalilipiran et al., 2020 [37], Iran (Cross-sectional)	Only the MD was associated with reduced CVD risk and sudden cardiac death The DASH diet led to lower levels of total cholesterol, triglycerides, LDL-C, HDL-C, and LDL/HDL ratio; the MD only improved LDL/HDL ratio The DASH diet improved insulin, hs-CRP, fibrinogen levels, and DBP, while the MD only improved DBP and fibrinogen levels
Plant-based (Vegetarian/Vegan)	Excludes meat and meat products, poultry, and fish/seafood Variety of plant foods, such as fruits, vegetables, legumes, and whole grains	High in dietary fibre; phytochemicals; antioxidants; and minerals such as magnesium, folic acid, and vitamin C Low in cholesterol and total fat	Lipid-lowering effect Lower BP Reduced BMI levels Lower incidence of CVD	Sofi et al., 2018 [38], Italy (RCT) Rogerson et al., 2018 [39], UK (RCT) Barnard et al., 2020 [40], US (Cross-sectional RCT)	Both the MD and the vegetarian diet led to significant reductions in BMI, fat mass, and body weight, and there were no significant group differences between the two diets The MD was more beneficial to vasodilatory ability and NO levels than the vegan diet The vegan diet led to greater improvements in body weight, insulin sensitivity, and lipid concentrations than the MD The MD led to greater improvements in BP
Nordic	Vegetables and fruits (mainly root vegetables, apples/pears, and berries), whole grains (e.g., rye, barley), seafood/fish, and use of rapeseed oil	Higher in dietary fibre, phytochemicals, antioxidants, and monounsaturated fats	Decrease in body weight and DBP Improved lipid profile Lower incidence of CVD and stroke	Bonaccio et al., 2021 [41], Italy (prospective cohort) Galbete et al., 2018 [42], Germany (prospective cohort) Li et al, 2015 [43], Sweden, (prospective cohort)	The MD lowered the risk of all-cause mortality and T2DM, but the Nordic diet showed no significance for either outcome
Japanese-style diets	Fruit, vegetables, legumes (mainly soybean/soy products), rice bran oil as the primary source of fat, fermented foods such as miso soup and pickles, seafood, seaweed, and rice Lower in animal protein, particularly red meat	Lower in total fat Increased flavonoids and dietary fibre	Decrease in BP, body weight, and BMI Improved LDL and HDL cholesterol levels Lower incidence of CVD and stroke mortality	N/R	N/R

CVD, cardiovascular disease; DASH, dietary approaches to stop hypertension; DBP, diastolic blood pressure; HDL-C, high-density lipoprotein cholesterol; hs-CRP, high-sensitivity C-reactive protein; LDL-C, low-density lipoprotein cholesterol; LF, low-fat; N/R, not reported; MD, Mediterranean diet; RCT, randomized–controlled trial; SBP, systolic blood pressure; SR, systematic review; T2DM, type 2 diabetes mellitus; US, United States of America.

**Table 2:** Characteristics of interventional and observational studies with sex-disaggregated or female-specific populations.

Author, Year, Country	Study Design	Population	Sample Size		Study Duration/ Follow-Up	Intervention/ Exposure	Comparator	Outcome	Result: MD vs. Comparator
			N	% Female					
CVD									
Fung et al., 2009 [70] (NHS I), US	Prospective cohort	Healthy female nurses without history of CVD (aged 38–63 years)	74,886	100%	20 years (maximum)	Higher MD adherence, assessed by an Alternate MDS	Lower MD adherence	CHD Stroke	Higher MD adherence led to: Lower CHD incidence, RR = 0.71 (95% CI 0.62–0.82) Lower stroke incidence, RR = 0.87 (95% CI 0.73–1.02)
Estruch et al., 2018 [31] (PREDIMED), Spain	RCT	Participants without CVD (aged 55–80 years)	7447	57%	Median follow-up of 4.3 years	MD with EVOO or nuts	Low-fat	MACE (defined as myocardial infarction, stroke, or death from cardiovascular causes)	Exposure to the two MD groups combined led to: Lower risk of MACE in men only (HR = 0.69 (95% CI 0.51–0.94)), but not in women (HR = 0.73 (95% CI 0.50–1.07)) No sex interaction = *p* = 0.62
Delgado-Lista et al., 2020 [32] (CORDIOPREV), Spain	RCT	Patients with CHD (aged 20–75 years)	1002	17.5%	7 years follow-up	MD rich in olive oil	Low-fat	MACE	Higher MD adherence led to: Lower MACE risk for men (HR = 0.68 (95% CI 0.50–0.94)) but not for women (HR = 1.27 (95% CI 0.64–2.49)). Sex interaction: *p* = 0.03
Rosato et al., 2019 [10]	SR of 29 observational studies	Participants without CVD (aged ≥18 years)	N/A	N/A	N/A	Higher MD adherence, assessed using MDS	Lower MD adherence	Overall CVD	Higher MD adherence led to: Lower CVD risk for women (RR = 0.85 (95% CI 0.72–0.98)) and men (RR = 0.85 (95% CI 0.72–0.98))
Pant et al., 2023 [11]	SR of 16 prospective cohort studies	Participants without CVD (aged ≥18 years)	722,495	100%	Median follow-up of 12.5 years	Higher MD adherence, assessed using MDS	Lower MD adherence	Incident CVD Total mortality	Higher MD adherence led to: Lower CVD incidence in women: HR = 0.76 (95% CI 0.72 to 0.81) Total mortality HR = 0.77 (95% CI 0.74 to 0.80) Lower CVD incidence in men HR = 0.78 (95% CI 0.72 to 0.83) Total mortality HR = 0.77 (95% CI 0.75 to 0.79)
Tang et al., 2021 [23]	SR of 7 cohort studies	Participants with history of CVD	37,879	N/A	Between 3.8 to 10.0 years	Higher MD adherence, assessed using MDS	Lower MD adherence	Total mortality	Women: no significant effect (HR = 0.97 (95% CI 0.92–1.02)) Men: inverse association with total mortality (HR = 0.94 (95% CI 0.90–0.98))
Traditional cardiovascular risk factors									
Esposito et al., 2003 [71], Italy	RCT	Premenopausal women with obesity (aged 20–46 years)	120	100%	2 years	MD intervention: education on dietary calories, personal goal setting, and self-monitoring	Usual care	Anthropometric parameters, BP Insulin sensitivity Lipid profile Inflammatory markers	In the MD group, significant reductions in body weight, BMI, SBP/DBP, glucose, insulin and HOMA, triglycerides, and FFA. HDL-C was increased more significantly in the intervention. Serum concentrations of interleukins 6 and 18 and hs-CRP were significantly reduced.
Toobert et al., 2003 [72], US	RCT	Postmenopausal women with T2DM (aged >30 years)	279	100%	6 months	Mediterranean lifestyle program: MD, stress management, exercise, and smoking cessation	Usual care	HbA1c Lipid profile BMI BP Plasma fatty acids	In the MLP group, significant improvements were observed for HbA1c, BMI, plasma fatty acids, and quality of life at the 6-month follow-up
Storniolo et al., 2017 [73], Spain (PREDIMED)	RCT	Women with moderate hypertension (aged 60–80 years)	90	100%	1 year	MD with EVOO or nuts	Low-fat	Endothelial markers: nitric oxide and endothelial-1	Improvements in endothelial markers for both the MD interventions, but not the low-fat diet
Dunn et al., 2014 [74], Australia	RCT	Premenopausal women who were overweight (mean age 22 ± 0.8 years)	30	100%	12 weeks	Combined lifestyle intervention: high-intensity intermittent exercise, MD, and fish oil	Usual care	Anthropometric parameters Insulin resistance Inflammatory markers Blood pressure	In the MD-integrated lifestyle intervention, significant reductions in BMI, abdominal adiposity, waist circumference, SBP, fasting insulin, triglycerides and interleukin-6.
Buscemi et al., 2009 [75], Italy	RCT	Healthy women who were overweight/obese (aged 30–55 years)	20	100%	2 months	Mediterranean hypocaloric diet (group M)	Atkins low-carbohydrate diet (group A)	Endothelial function assessed by flow-mediated dilation Metabolic parameters Lipid profile	At follow-up, Group M had significantly greater reductions in SBP. More significant weight loss in Group A than Group M No group significance for endothelial function
Bajerska et al., 2018 [76], Finland	RCT	Postmenopausal women with central obesity	144	100%	16 weeks	MD	Central European diet	Body weight Visceral fat loss	Similar improvements in both groups, with group significance only for visceral fat in women consuming the Central European diet.
Bedard et al., 2012 [57], Canada	RCT	Men and premenopausal women (aged 25–50 years)	70	45.7%	4 weeks	Isoenergetic MD	N/A	Cardiometabolic parameters	Total cholesterol, LDL-C, and diastolic BP significantly decreased in both. Only men had significantly improved insulin homeostasis and SBP. More significant effects on body weight, BMI, and 2 h postload insulin were found in men
Jennings et al., 2009 [77], 5 recruitment centres in Europe: Italy, Netherlands, Poland, France, United Kingdom	RCT	Men and women aged 65–79 years	1294	48.3%	1 year	MD group received tailored standardised dietary advice administered 9 times (via telephone/in person)	Usual care	BP measurements Arterial stiffness assessed by pulse wave velocity	Significant reduction in SBP in males, but not females. Significant improvements in atrial stiffness in females, but not males. Significant interaction with sex and SBP.
Ahmad et al., 2020 [78], US	Prospective cohort study	Healthy women without baseline diabetes (mean age: 52.9 ± 9.9 years)	25,317	100%	Mean 19.8 years	Higher MD adherence, assessed by the Alternate MD	Lower MD adherence	T2DM	Lower risk of T2DM in the higher MD adherence group: HR = 0.85 (95% CI, 0.76–0.96)
Salas-Salvado et al., 2011 (corrected in 2018) [79], Spain (PREDIMED)	RCT	Non-diabetic men and women (aged 55–80) years	418	58.4%	Median 4.0 years	MD with EVOO or nuts	Low-fat	T2DM	Lower risk of T2DM only for female participants consuming the MD: MD with nuts vs. control Male: HR = 0.65 (95% CI, 0.21–2.00) Female: HR = 0.32 (95% CI 0.11–0.93) Both MD groups vs. control: Male: HR = 0.55 (95% CI 0.21–1.43) Female: HR = 0.40 (95% CI 0.18–0.90)
PCOS									
Mei et al., 2022 [80], China	RCT	Patients with PCOS who were overweight (aged 16–45 years)	72	100%	12 weeks	Low-carbohydrate MD	Low-fat	Anthropometric parameters, insulin resistance, and lipids	In the MD group, significant reductions in weight, BMI, body fat percentage, and HOMA-IR were found. Significant differences in total cholesterol, triglycerides, and LDL-C were observed in the low-fat group.
Cincione et al. [81], 2022, US	RCT	Women with PCOS who were overweight/ obese (aged 18–45 years)	144	100%	45 days	MD	Ketogenic diet	Anthropometric parameters	Significant reductions in all parameters in the ketogenic group compared to the MD group.
Pregnancy-related complications									
Perez-Ferre et al., 2014 [82], Spain	RCT	Women with past GDM 6 to 12 weeks postpartum	260	100%	3 weeks	Mediterranean lifestyle intervention on nutrition and physical activity	Control	Glucose disorders of impaired fasting glucose, impaired glucose tolerance, or DM2	The MD-integrated lifestyle program led to a reduction in all glucose disorders.
Reimer et al., 2021 [83], Germany	Prospective RCT	Women with past HDP 6-weeks postpartum	38	100%	6 months	MD and cardiovascular exercise program	Control	Arterial stiffness measured by pulse wave velocity	Significant reduction in arterial stiffness in the intervention group compared to control
Crovetto et al, 2020 [84], Spain Casas et al, 2023 [85], Spain	Parallel-group RCT	Pregnant individuals at 19–23 weeks’ gestation	1221	100%	34–36 weeks	MD group OR 8-week stress reduction program adapted for pregnancy	Usual care	Incidence of SGA maternal stress, well-being, and sleep quality	Significantly lower rates of SGA babies after delivery in both the MD and the stress reduction group Significantly lower maternal stress, anxiety, and sleep quality in the MD group than usual care
Inflammatory autoimmune diseases									
McKellar et al., 2007 [86], Scotland	Pilot RCT	Patients with RA (aged 30–70 years)	130	100%	6 weeks	Nutritional information on the Mediterranean-style diet provided in weekly 2 h sessions	Dietary written information only	Pain score Early morning stiffness SBP	Improvements in pain scores at 3 months and 6 months, early-morning stiffness at 6 months, and significant improvements in SBP in the intervention group only.
Papandreou et al., 2023 [87], Greece	RCT	Women with RA in remission (mean age 34.03 ± 5.45 years)	40	100%	12-week	Isocaloric MD plan with lifestyle consultations on physical activity	Control	Disease activity Anthropometric parameters Blood lipids	Greater improvements in the MD group than the control for body weight, body composition, blood glucose, and lower disease activity.
Pocovi-Gerardino et al., 2021 [88], Spain	Cross-sectional study	Patients with SLE (mean age: 46.9 ± 12.85 years)	280	90.4%	N/A	Higher MD adherence	Lower MD adherence	Anthropometric parameters, disease activity, inflammatory markers, cardiovascular risk factors	Patients consuming higher versus lower MD intake had significantly lower fat mass percentage, BMI, and triglycerides. Higher MD adherence was associated with less damage and disease activity.

BMI, body mass index; CI, confidence intervals; CVD, cardiovascular disease; DBP, diastolic blood pressure; GDM, gestational diabetes mellitus; HbA1C, glycated haemoglobin; HDL-C, high-density lipoprotein cholesterol; HDP, hypertensive disorders of pregnancy; hs-CRP, high-sensitivity C-reactive protein; HOMA-IR, homeostatic model assessment for insulin resistance; HR, hazard ratio; LDL-C, low-density lipoprotein cholesterol; MACE, major adverse cardiovascular events; MD, Mediterranean diet; MDS, MD score; N/A, not applicable; PCOS, polycystic ovarian syndrome; *RA*, rheumatoid arthritis; RCT, randomized–controlled trial; RR, risk ratio; SBP, systolic blood pressure; SLE, systemic lupus erythematosus; T2DM, type 2 diabetes mellitus; US, United States of America.

## Data Availability

Not applicable.

## Abbreviations

CVD	cardiovascular disease
MD	Mediterranean diet
EVOO	extra-virgin olive oil
GDM	gestational diabetes mellitus
PCOS	polycystic ovarian syndrome
RA	rheumatoid arthritis
SLE	systemic erythematous lupus
BP	blood pressure
ROS	reactive oxygen species
BMI	body mass index
DASH	dietary approaches to stop hypertension
LDL-C	low-density lipoprotein cholesterol
HDL-C	high-density lipoprotein cholesterol
Hs-CRP	high-sensitivity C-reactive protein
T2DM	type 2 diabetes mellitus
APOE	apolipoprotein E gene
ACE	angiotensin-converting enzyme
RCT	randomized–controlled trial
PREDIMED	Prevención con Dieta Mediterránea
CORDIOPREV	CORonary Diet Intervention with Olive oil and cardiovascular PREVention
US	United States of America
CHD	coronary heart disease
HDP	hypertensive disorders of pregnancy
LC-PUFA	long-chain polyunsaturated fatty acid
